# Balancing Privacy and Precision: Evaluating Meta-Analysis for National Health Data Integration in Canada

**DOI:** 10.23889/ijpds.v9i5.2882

**Published:** 2024-09-18

**Authors:** Megan Harmon, Jason Black, Na Li, Tolulope Sajobi, Jessalyn Holodinsky, Tyler Williamson

**Affiliations:** 1 University of Calgary

## Background

In Canada, legislative restrictions exist for the disclosure and transfer of patient data across provincial boundaries. Often, meta-analysis techniques are employed to pool province-specific results to enable national results while maintaining compliance with privacy legislation. However, the effectiveness of this technique remains unexamined using electronic data. To evaluate its performance, we compare results obtained through meta-analysis with those from a fully pooled model.

## Methods

Using chronic kidney disease as a case study, this retrospective cohort study simulates the meta-analysis method to evaluate its performance using real data. We analyze data from the Canadian Primary Care Sentinel Surveillance Network (CPCSSN), a nationwide database of electronic medical records. We meta-analyze provincial logistic regression models to predict nephropathy over a 5-year period at a national level. Estimates are compared to a fully pooled national model.

## Results

Analyses are currently ongoing. We expect the meta-analysis technique to produce similar estimates compared to the fully pooled model. However, we expect certain differences as meta-analysis techniques are insensitive to heterogeneity within provinces that impact the precision of estimates. Observed differences will help to inform future work where we will examine a potential new methodology to analyze health information without sharing patient data.

## Conclusions

Based on our results, we will reveal strengths and limitations of the meta-analysis technique for interprovincial analyses. Limitations of the meta-analysis technique may underscore the need for further exploration into alternative methodologies that can effectively analyze health information without compromising patient privacy to facilitate better healthcare decision-making and policy development in Canada.

